# Exploring Tau-C, Tau-A, and C1M as biomarkers for Parkinson’s disease

**DOI:** 10.3389/fneur.2025.1566228

**Published:** 2025-04-30

**Authors:** Meryem Benmarce, Kim Henriksen, Morten Karsdal, Signe Holm Nielsen

**Affiliations:** ^1^Nordic Bioscience, Herlev, Denmark; ^2^Department of Health Technology, Technical University of Denmark, Lyngby, Denmark; ^3^Department of Molecular and Medical Biology, Roskilde University Center, Roskilde, Denmark

**Keywords:** biomarker, Parkinson’s disease, Tau-C, Tau-A, C1M

## Abstract

Tau has shown to be associated with neurodegenerative diseases. In this exploratory study, we investigated the utility of blood-based biomarkers measuring Tau-C, Tau-A, and C1M in Parkinson’s disease (PD) to explore their potential as diagnostic biomarkers in two independent cohorts (TAU-C and TAU-A). We found them to discriminate between healthy donors and PD individuals (all *p* < 0.001), with Tau-C showing the best diagnostic discrepancy (AUROC = 0.97). Therefore, Tau-C, Tau-A, and C1M may be biomarkers for PD diagnosis.

## Short communication

Parkinson’s disease (PD) is a progressive and irreversible neurodegenerative disease, affecting 6 million people worldwide (1). Clinically it is characterized by both motor and nonmotor symptoms. The main motor symptoms of PD include bradykinesia, stiffness, resting tremor, and postural instability. Nonmotor symptoms, which may manifest earlier and have a significant impact on the quality of life, encompass depression, constipation, and sleep disorders (2). At a molecular level PD is characterized by dopaminergic deficiency and protein aggregation (3).

Motor symptoms in PD are due to the dopaminergic hypofunction in the substantia nigra pars compacta therefore, the diagnosis of PD relies on symptoms linked to dopamine deficiency. However, it is clinically diagnosed by the evaluation of the patient’s medical history, poor sleep, loss of smell, constipation as well as the family history and ethnicity. Consequently, the difficulty of clinically diagnosing PD and the lack of a current diagnostic test frequently result in misdiagnosis (4). This underscores the importance of developing a blood biomarker (5). There is an increasing interest for non-invasive methods for early detection of PD since dopaminergic dysfunction precedes clinical symptoms. This is where molecular biomarkers become relevant; they have the potential to differentiate PD from other neurodegenerative disorders, track the progression of the disease, and serve as indicators to evaluate treatment effectiveness (6).

Treating PD is also challenging due to the limited ability of treatments to cross the blood-brain barrier (5). Currently there are different treatment options such as levodopa, COMT (catechol-O-methyltransferase) inhibitors and dopamine agonist (1). Those therapies improve motor symptoms however, they only target symptoms without slowing down the disease progression. Unfortunately, there are no drugs that prevent or slow down the disease (5) nevertheless, different drugs are currently under development to overcome the limitations of traditional medications (1); however, these would benefit from better and more easily accessible biomarkers.

Patients with PD disease is also characterized by protein aggregation of α-synuclein (α-Syn) in Lewy bodies and Lewy neurites deposits. Under physiological conditions, α-Syn is a soluble unfolded protein. However, during PD, its conformation changes to β-sheet oligomers, which then convert to amyloid fibrils and form Lewy bodies and Lewy neurites (7).

Previous research has indicated that α-Syn protein in PD is co-localized with Tau, a pathological constituent commonly found in Alzheimer’s disease (AD) (8). Tau is a protein expressed in neurons, but it can also be found in astrocytes and oligodendrocytes (9), its role is to stabilize microtubules (10). According to Vassili et al. (11), α-Syn and Tau exhibit prion-like characteristics, including the ability to misfold, seed, and spread the misfolded conformation to their respective monomeric forms. On a genetic level, utilizing genome-wide association study (GWAS) has shown that the gene responsible for encoding Tau, known as MAPT, is a risk gene for PD (12). In a study by Kang et al. (13), they discovered a strong correlation between the levels of Tau and α-Syn in the cerebrospinal fluid (CSF) of early-stage PD patients. These findings suggest that Tau plays a role in the pathogenesis of PD.

Another noteworthy pathological mechanism to investigate is the proteolytic cleavage of Tau by several proteases, such as caspases, which have been shown to initiate neuronal cell death. Caspase-3 cleaves Tau at a specific site (Asp361) resulting in fragments known as Tau-C, which are suggested to facilitate nucleation-dependent filament formation (14, 15). Another protease, ADAM10, has a significant role in neuronal degeneration and generates the Tau-A fragment, which is correlated with cognitive function in AD. These fragments can pass through the blood-brain barrier likely due to their small size and can be detected in serum (15). Since hyperphosphorylated Tau induces α-Syn aggregation and the formation of Lewy body (10), another post translational modification such as truncation may also contribute to α-Syn aggregation. Moreover, research has shown that neo-epitopes fragments can be useful biomarkers for neurodegeneration (15). It is therefore suggested that Tau-C and Tau-A could be associated to neuronal degeneration in PD.

Moreover, it is suggested that neuroinflammation might play an important role in PD pathogenesis due to the increased levels of inflammatory biomarkers in PD patients (16). Studies show that activated microglia by inflammogens induces caspase-3/7 activation, and inhibiting these caspases halts microglial activation and neuroinflammation (17). Furthermore, C1M a biomarker that measures serum levels of matrix metalloproteinase (MMP)-degraded type I collagen, which reflects cell inflammation has also been correlated with Tau-C and Tau-A in demented/AD patients. It was suggested that uncontrolled inflammation might lead to the release of degraded tau in the periphery (18).

With these findings in mind, the objective of this study was to investigate the potential of the neurodegeneration biomarkers Tau-C, Tau-A and the neuroinflammation biomarker C1M as diagnostic biomarkers for PD.

The discovery cohort included serum samples from patients with PD (*n* = 16) and healthy donors (*n* = 11), the evaluation cohort included PD patients (*n* = 30) and healthy donors (*n* = 15). Both cohorts were obtained from Proteogenex (Culver City, CA, United States), following standardized protocols, and stored at −80°C until biomarker evaluation. Written informed consent was obtained from all donors, and the study was approved by the local ethics committee, adhering to the principles outlined in the Helsinki Declaration of 2013.

The discovery cohort consisted of 16 PD patients (8 male, 8 female), with a mean age of 73.7 years (SD = 6.7), and 11 healthy donors (7 male, 4 female), with a mean age of 70.8 years (SD = 1.25). The evaluation cohort contained 30 PD patients (8 male, 8 female), with a mean age of 64.2 years (SD = 6.7), and 15 healthy donors (9 male, 6 female), with a mean age of 60.3 years (SD = 5.6) (Table 1). Using competitive ELISA assays, we examined the ability of Tau-C, Tau-A, and C1M to distinguish PD patients from healthy donors. The inter-assay and intra-assay were found to be less than 15 and 10%, respectively.

**Table 1:** Demographic table.

Characteristics	Discovery cohort			Evaluation cohort		
	Healthy donors (*n* = 11)	Parkinson’s disease (*n* = 16)	*p*-value	Healthy donors (*n* = 15)	Parkinson’s disease (*n* = 30)	*p*-value
Age, mean (SD)	70.8 (1.25)	73.7 (2.89)	0.005	60.3 (5.6)	64.2 (6.7)	0.065
Sex, male, *n* (%)	7 (63.6%)	8 (50%)		9 (60.0%)	15 (50%)	0.531
BMI, mean (SD)	25.8 (2.2)	25.2 (3.7)	0.592	NA	26.1 (3.2)	
Caucasian, *n* (%)	11 (100%)	16 (100%)	1.000	15 (100%)	30 (100%)	1.000
Years since diagnosis, mean (SD)	NA	1.5 (2.0)		NA	1.1 (0.4)	
Signs						
Hypokinesia, *n* (%)		5 (31.3%)			8 (26.7%)	
Postural instability, *n* (%)		9 (56.3%)			10 (33.3%)	
Muscle rigidity, *n* (%)		6 (37.5%)			14 (46.7%)	
Tremor, *n* (%)		9 (56.3%)			16 (53.3%)	
Current treatment						
Levodopa, *n* (%)		8 (50%)			10 (33.3%)	

BMI, body mass index. Categorical variables are presented as numbers (percentages), while continuous variables are reported as means (standard deviations). Statistical differences between the clinical and patient demographics were calculated using a Mann–Whitney test.

**Figure 1:**
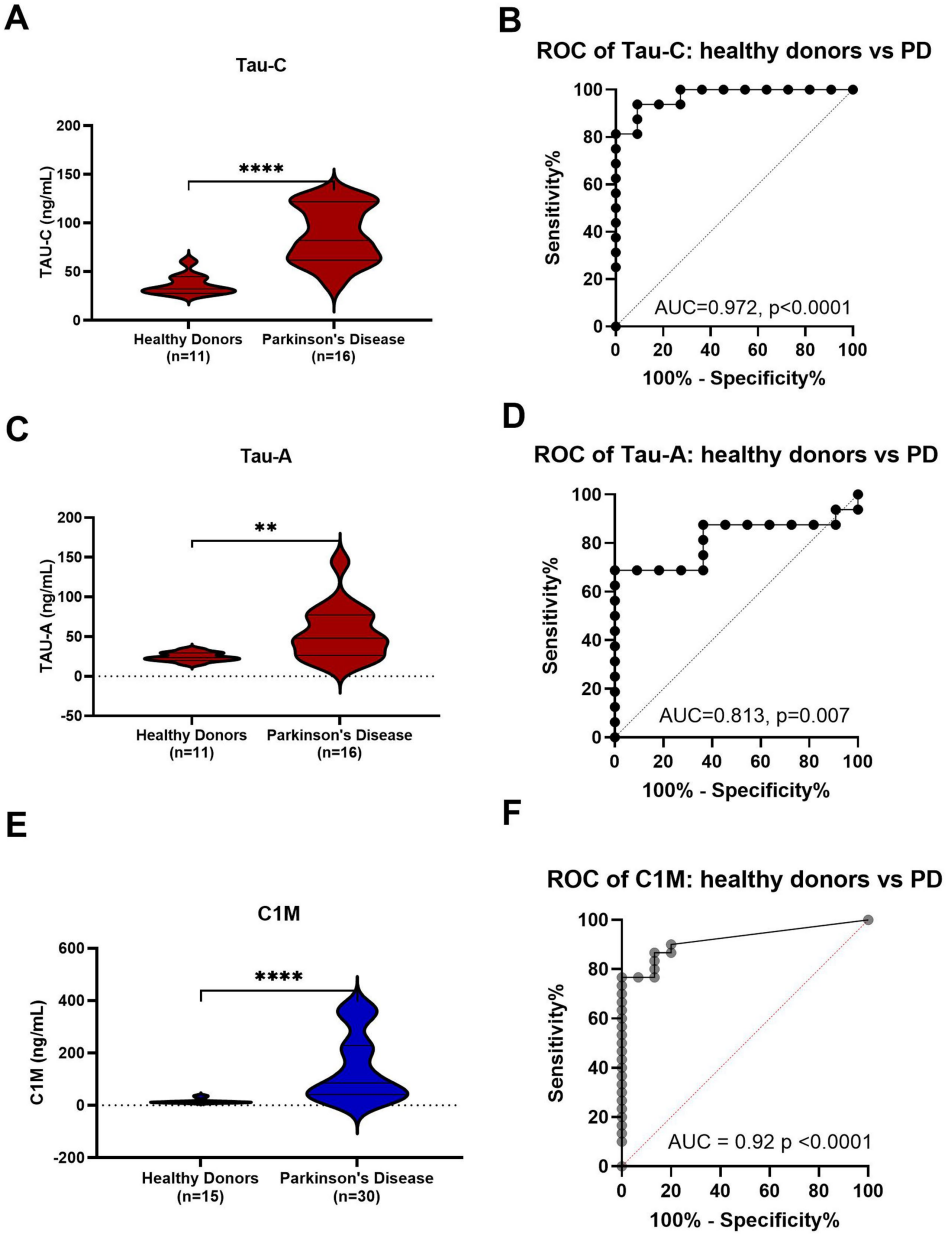
Levels of Tau-C and Tau-A in serum from healthy donors and patients with PD. **(A)** Tau-C in serum from healthy donors (*n* = 11) and PD (*n* = 16). **(B)** Receiver operating characteristics (ROC) curve analysis, evaluating the ability of Tau-C to discriminate between healthy donors and PD. **(C)** Tau-A in serum from healthy donors (*n* = 11) and PD (*n* = 16). **(D)** ROC curve analysis, evaluating the ability of Tau-A to discriminate between healthy donors and PD. **(E)** C1M in serum from healthy donors (*n* = 15) and PD (*n* = 30). **(F)** ROC curve analysis, evaluating the ability of C1M to discriminate between healthy donors and PD. Data were analyzed using a Mann–Whitney test or a ROC curve analysis. Data are presented as Tukey box plots. Significance levels: ^**^p < 0.01 and ^****^p < 0.0001.

**Figure 2:**
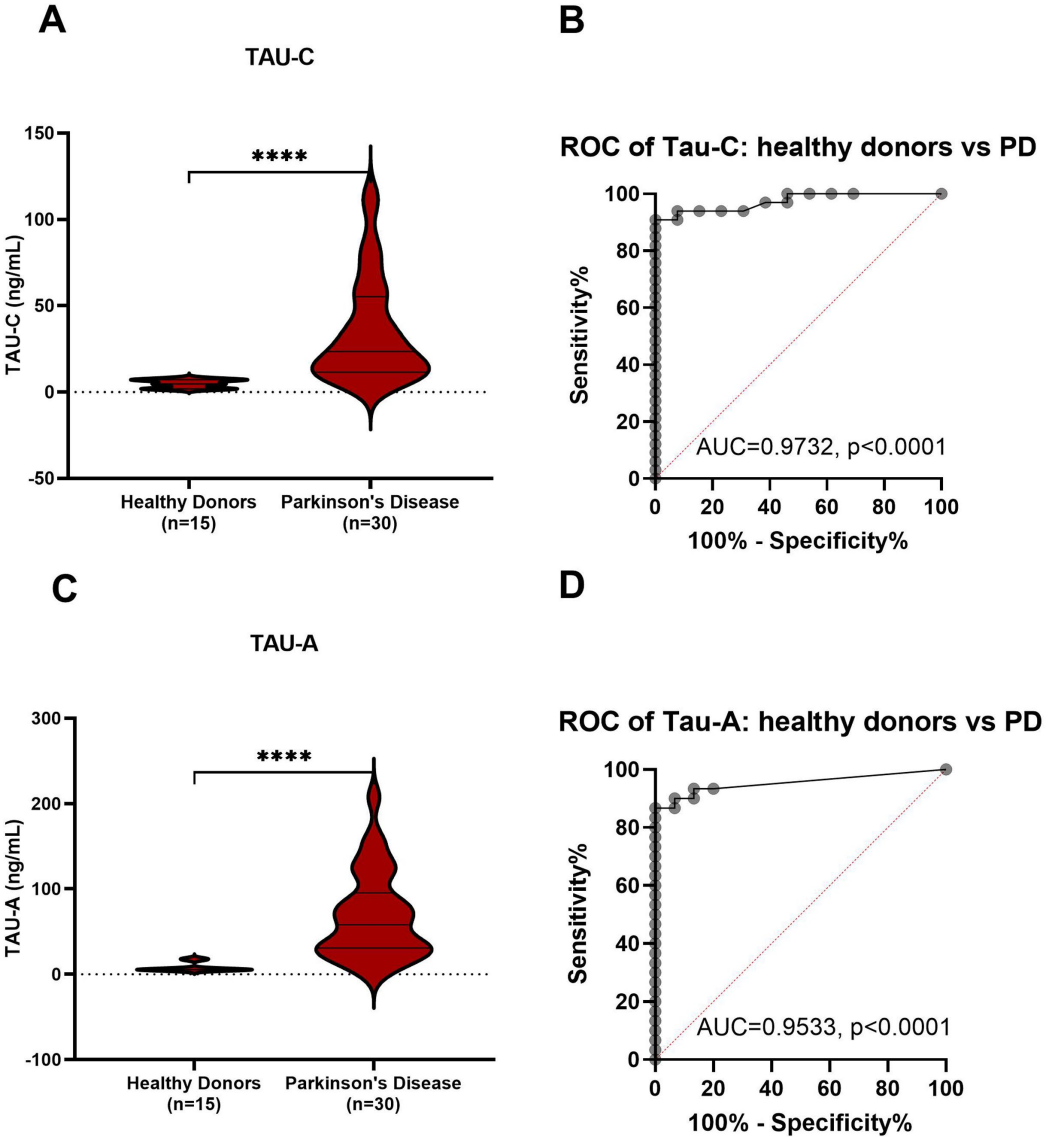
Levels of Tau-C and Tau-A in serum from healthy donors and patients with PD. **(A)** Tau-C in serum from healthy donors (*n* = 15) and PD (*n* = 30). **(B)** ROC curve analysis, evaluating the ability of Tau-C to discriminate between healthy donors and PD. **(C)** Tau-A in serum from healthy donors (*n* = 15) and PD (*n* = 30). **(D)** ROC curve analysis, evaluating the ability of Tau-A to discriminate between healthy donors and PD. Data were analyzed using a Mann–Whitney test or a ROC curve analysis. Data are presented as Tukey box plots. Significance levels: ^**^p < 0.01 and ^****^p < 0.0001.

Both Tau-C and Tau-A levels were significantly higher in patients with PD compared to healthy controls (*p* < 0.0001; Figure 1A and *p* < 0.01; Figure 1C, respectively). Diagnostic accuracy was evaluated by an area under the receiver operating characteristic curve (AUROC). The AUROC for Tau-C was 0.97 (95% CI: 0.92–1.00, *p* < 0.0001, Figure 1B), slightly surpassing the AUROC for Tau-A 0.81 (95% CI: 0.64–0.98, *p* = 0.007, Figure 1D). The data was further validated using another cohort of 15 healthy donors and 30 PD (see Table 1), Tau-C and Tau-A consistently demonstrated a significant ability to distinguish between healthy controls and PD with an AUROC of 0.97 (95% CI: 0.93–1.00, *p* < 0.0001) and 0.95 (95% CI: 0.89–1.00, *p* < 0.0001) respectively (see Figure 2).

Furthermore, this study demonstrates that the previously validated C1M biomarker, which was found to be correlated with Tau-C and Tau-A in patients with dementia/AD (18), exhibits higher levels in PD compared to healthy donors (*p* < 0.0001; Figure 1E) with an AUROC of 0.92 (95% CI: 0.84–0.99, *p* < 0.0001 Figure 1F). This observation suggests that the elevated levels of C1M in PD may lead to the release of degraded tau to the periphery due to uncontrolled inflammation. These findings further support the potential utility of Tau-C and Tau-A as biomarkers for PD.

We evaluated the biomarkers Tau-C and Tau-A and the neuroinflammation biomarker C1M, quantifying the area under the receiver operating characteristic curve in patients with PD, with the aim of distinguishing PD patients from healthy individuals. PD does not have a recognized and reliable biomarker to implement as diagnostic, prognostic, or efficacy of intervention tools. The development of such biomarkers is important to allow for intervention before the disease becomes symptomatic, and secondly, to enable monitoring of the effectiveness of potential therapies that could slow down or prevent disease progression. Personalized medicine shows promise in predicting, individually, patient’s susceptibility to developing specific diseases. This pilot study shows that Tau-C, Tau-A, and C1M may be biomarkers for PD diagnosis However, the limited sample size necessitates further validation with a larger population. Moreover, comparing the levels of these biomarkers across various neurodegenerative diseases may enhance the diagnostic accuracy for PD, particularly considering that tau fragments and C1M levels may escalate during neurodegenerative processes.

In conclusion, this is the first study to quantitatively measure Tau-C, Tau-A, and C1M levels in serum from PD patients and demonstrate their clinical utility as potential diagnostic tools. Such biomarker could provide valuable information for assessing patients’ suitability for targeted treatments, thereby addressing the current gap in biomarkers for clinical management and trials. Moreover, other approaches, such as the analysis of saliva, tears, and CSF show promise; but require further studies.

## Data Availability

The raw data supporting the conclusions of this article will be made available by the authors, without undue reservation.
